# Maintenance tegafur-uracil versus observation following an adjuvant oxaliplatin-based regimen in patients with stage III colon cancer after radical resection: study protocol for a randomized controlled trial

**DOI:** 10.1186/s13063-017-1904-9

**Published:** 2017-04-26

**Authors:** Yung-Sung Yeh, Hsiang-Lin Tsai, Ching-Wen Huang, Po-Li Wei, Yung-Chuan Sung, Hsiu-Chih Tang, Jaw-Yuan Wang

**Affiliations:** 1 Division of Trauma and Critical Care, Department of Surgery, Kaohsiung Medical University Hospital, Kaohsiung Medical University, Kaohsiung, Taiwan; 2 Division of Colorectal Surgery, Department of Surgery, Kaohsiung Medical University Hospital, Kaohsiung Medical University, Kaohsiung, Taiwan; 3 Department of Emergency Medicine, Kaohsiung Medical University Hospital, Kaohsiung Medical University, Kaohsiung, Taiwan; 4 Graduate Institute of Clinical Medicine, College of Medicine, Kaohsiung Medical University, Kaohsiung, Taiwan; 5 Division of General Surgery Medicine, Department of Surgery, Kaohsiung Medical University Hospital, Kaohsiung Medical University, Kaohsiung, Taiwan; 6 Department of Surgery, Faculty of Medicine, College of Medicine, Kaohsiung Medical University, Kaohsiung, Taiwan; 7 Graduate Institute of Medicine, College of Medicine, Kaohsiung Medical University, Kaohsiung, Taiwan; 8 Division of Colorectal Surgery, Department of Surgery, Taipei Medical University Hospital, Taipei Medical University, Taipei, Taiwan; 9 Colon and Rectal Surgery, Tainan Sin-Lau Hospital, Tainan, Taiwan; 10 Division of Hematology-Oncology, Department of Internal Medicine, Cathay General Hospital, Taipei, Taiwan; 11 Center for Biomarkers and Biotech Drugs, Kaohsiung Medical University, Kaohsiung, Taiwan; 12 Research Center for Environmental Medicine, College of Medicine, Kaohsiung Medical University, Kaohsiung, Taiwan; 13 Research Center for Natural Products and Drug Development, Kaohsiung Medical University, Kaohsiung, Taiwan; 14 Division of Colorectal surgery, Department of Surgery, Faculty of Medicine, College of Medicine, No. 100 Tzyou 1st Road, San-Ming District, Kaohsiung, 807 Taiwan; 15 Department of Surgery, Kaohsiung Medical University Hospital, Kaohsiung Medical University, No. 100 Tzyou 1st Road, San-Ming District, Kaohsiung, 807 Taiwan

**Keywords:** Tegafur-uracil, Oxaliplatin, Stage III colon cancer

## Abstract

**Background:**

We conducted a prospective randomized study of an adjuvant oxaliplatin-based regimen plus orally administered tegafur-uracil in patients with stage III colon cancer after radical resection to evaluate the feasibility of this drug combination in cancer clinical outcomes, acute toxicity, disease-free survival (DFS), and overall survival (OS) in Taiwan.

**Methods/design:**

This is an open-label, randomized, comparative, double-arm, multicenter, phase III study to assess DFS, OS, and safety profiles of the aforementioned drug combination as maintenance therapy for 1 year in patients with stage III colon cancer after radical resection in Taiwan. Following the completion of an adjuvant oxaliplatin-based regimen for 3 weeks with no evident disease recurrence, all eligible patients will be randomly assigned to either arm A (maintenance therapy) or arm B (observation arm) in a 2:1 ratio (364 and 182 patients in the tegafur-uracil and observation groups, respectively). Treatment in arm A will be started within 7 days of randomization. If the patients reported disease recurrence, intolerable toxicity, withdrew consent or the investigator determined that the patient should be withdrawn during the study period, they were withdrawn from the study. If a patient was discontinued from the study, the corresponding data were not reused, and the patient was not allowed to re-enter the study.

**Discussion:**

A unique characteristic of this intervention was that the adjuvant chemotherapy with oxaliplatin and tegafur-uracil was anticipated to be safe and has high treatment efficacy, with the advantage of yielding a favorable response rate and tolerable toxicity profile.

**Trial registration:**

ClinicalTrials.gov, identifier: NCT02836977. Registered on 18 July 2016.

**Electronic supplementary material:**

The online version of this article (doi:10.1186/s13063-017-1904-9) contains supplementary material, which is available to authorized users.

## Background

### Epidemiology

Colorectal cancer (CRC), the most frequently diagnosed cancer, is the third leading cause of cancer-related deaths in Taiwan. During the past decade, the incidence rate of CRC has increased from 38 to 70 per 100,000 men and 30 to 51 per 100,000 women. The annual incidence of CRC in Taiwan has increased up to 43.5% in the past decade, with more than 15,000 new cases and >5000 deaths annually; among these patients, approximately one fourth had stage III disease according to the Taiwan Cancer Registry Database [1, 2]. Approximately 42% of stage III colon cancer cases have recurrences within 8 years after radical resection. In the 8-year follow-up period, 82% of patients with stage III colon cancer experienced recurrences in the initial 3 years [3].

### Current treatment modality

Intravenously administered (IV) 5-fluorouracil (FU) has been the most widely used chemotherapeutic agent for CRC for more than 40 years [4] and is included in various combination treatments such as those with oxaliplatin and irinotecan with or without biological agents (i.e., cetuximab and bevacizumab) [5]. Since 2004, FOLFOX4 (85 mg/m^2^ oxaliplatin combined with 200 mg/m^2^ leucovorin (LV) over 2 h, followed by a 400-mg/m^2^ 5-FU bolus and 22-h continuous infusion of 600 mg/m^2^ 5-FU) has been the accepted first-line therapy in advanced CRC [6]. The FOLFOX6 regimen comprises 100 mg/m^2^ oxaliplatin and 400 mg/m^2^ LV as a 2-h infusion on day l, followed by a 400-mg/m^2^ 5-FU IV bolus and a 46-h continuous infusion of 2400–3000 mg/m^2^ 5-FU; this regimen was repeated at 2-week intervals.

### Investigational product (IP) description

Tegafur-uracil, containing tegafur and uracil in a molar ratio of 1:4, is an antimalignant tumor agent with an antimetabolic effect. Tegafur is metabolized in vivo to 5-FU, which is a pyrimidine analog antimetabolite that is metabolized to 5-fluoro-2’-deoxyuridine monophosphate (FdUMP) and 5-fluorouridine triphosphate (FUTP). FdUMP inhibits deoxyribonucleic acid (DNA) synthesis by binding to thymidylate synthase and inhibiting thymidylate production. FUTP interferes with ribonucleic acid (RNA) processing when it is mistakenly incorporated instead of uridine triphosphate. Uracil facilitates maintaining intracellular levels of 5-FU by inhibiting its degradation.

Clinical trials have confirmed that the optimal combination ratio of tegafur-uracil yields long-lasting characteristics and high 5-FU concentrations in tumors. They have revealed the efficacy of tegafur-uracil as an antitumor agent for treating head and neck, gastric and breast cancers and CRC.

### Preclinical data

Tegafur-uracil can inhibit the growth of tumors such as Walker 256 carcinosarcoma, Yoshida sarcoma, ascites carcinoma (in rats), sarcoma 180, Ehrlich carcinoma, Lewis lung carcinoma, and B-16 melanoma (in mice) transplanted subcutaneously. It can also inhibit the growth of human cancers, such as gastric, beast, and pancreatic cancers, when transplanted subcutaneously in nude mice. In addition, its survival effects have been proven in animals (mice) bearing L1210 transplanted leukemia.

The antitumor activity of tegafur-uracil is based on 5-FU that appears gradually in the body through the transformation of tegafur. The mechanism of 5-FU is considered to be the inhibition of DNA synthesis, resulting from the antagonistic effect of the active metabolite FdUMP on dUMP to inhibit thymidylate synthase, and RNA-function disorders, resulting from the incorporation of FUTP into RNA. Uracil enhances the antitumor activity of tegafur.

### Clinical data and experience

The efficacy of adjuvant treatment in CRC was clearly demonstrated as recently as the early 1990s, and 5-FU + LV became the standard treatment for stage III colon cancer in 1996 [7, 8]. The MOSAIC trial [9] demonstrated the superiority of FOLFOX4 over 5-FU + LV, and in 2004, the FOLFOX4 regimen became the standard adjuvant treatment for stage III colon cancer.

Several clinical trials have demonstrated the efficacy of tegafur-uracil in postoperative adjuvant chemotherapy. The basic data from these studies indicate a possible contribution of the antiangiogenic activity of tegafur-uracil to its overall antitumor activity, which until now has been considered to be mediated by the cytotoxic effects of 5-FU. Thus, tegafur-uracil seems to be particularly useful in a chronic postoperative adjuvant chemotherapy regimen to prevent cancer progression.

Each cycle of tegafur-uracil + LV reduced the mean clinical visits by 66%, house staff visits by 17%, health professional visits by 36%, transfusion procedures by 74%, and diagnostic procedures by 33% compared with 5-FU + LV. In addition, tegafur-uracil + LV reduced monthly clinical visits of each patient by 4.5 h compared with 5-FU + LV. Tegafur-uracil + LV was associated with lower resource use than was an 5-FU + LV IV bolus, mainly because of fewer hospitalizations for the treatment of adverse events (AEs; tegafur-uracil + LV (21%) versus 5-FU (36%)) [10].

### Trial rationale

Additional maintenance therapy (12 cycles of tegafur-uracil (400 mg daily) + LV ((60 mg daily) for 28 days at a 7-day interval), followed by six cycles of 5-FU (375 mg/m^2^) + LV (30 mg daily) by a rapid IV injection for 5 days every 4 weeks (the Mayo Clinic regimen) significantly enhanced disease-free survival (DFS) compared with 5-FU + LV in patients with stage III colon cancer [11]. In patients with advanced CRC, a sequential therapy with a highly active chemotherapeutic regimen (e.g., FOLFOX4) for 6 months, followed by maintenance therapy with orally administered fluoropyrimidine (e.g., tegafur-uracil) is advantageous for obtaining a favorable toxicity profile and ease of administration by the oral route which is preferable for patients because of the more convenient outpatient therapy. The maintenance therapy with oral tegafur-uracil for patients responding to FOLFOX4 can maintain the treatment response and improve quality of life because it allows an outpatient regimen and the oral therapy entails lower psychological distress [5].

Adjuvant chemotherapy may prolong the 3-year DFS in patients with stage III colon cancer. However, cold-triggered, acute sensory reversible neuropathy and treatment-limiting chronic sensory neurotoxicity are associated with the cumulative dose of oxaliplatin [12], which is an indication to stop oxaliplatin treatment in patients who are still responding to treatment. Therefore, we designed a trial to demonstrate the utility of maintenance therapy with tegafur-uracil in patients with stage III colon cancer after a 6-month oxaliplatin-based regimen. The primary objective of this trial was to verify the 3-year DFS after 1 year of tegafur-uracil treatment.

## Objective

### Primary objective

To compare the 3-year DFS of tegafur-uracil following an adjuvant oxaliplatin-based regimen with that of an adjuvant oxaliplatin-based regimen in patients with stage III colon cancer after radical resection.

### Secondary objectives

1. To assess and compare the 5-year overall survival (OS) in maintenance therapy and observation arms
2. To assess and compare the safety profiles in both arms

## Methods/design

We will recruit participants from following centers:

Kaohsiung Medical University Hospital (IRB number: KMUHIRB-F(I)-20160016), Cathay General Hospital, Taipei Medical University Hospital, Far Eastern Memorial Hospital, Tri-Service General Hospital, Taichung Veterans General Hospital, China Medical University Hospital, Chung Shan Medical University Hospital, Kaohsiung Veterans General Hospital, National Cheng Kung University Hospital.

## Patient selection and enrollment

### Patient number

Eligible patients will be randomized in two arms in a ratio of 2:1 to reach an approximate total of 546 patients. The sample size ratio is 2:1 (study arm is 2, control is 1), treatment duration is 1 year. Based on data of the 3-year DFS rate from our previous retrospective study, the proportion of control arm is 0.55 (55%) and of the study arm is 0.7 (70%) with a significance level of 0.05 and a power of 80%.

### Inclusion criteria

For inclusion in the study, each patient must fulfill the following criteria:

1. Pathology-confirmed colon carcinoma
2. Stage III disease (T_1–4_, N_1–2_, and M_0_, as defined by the American Joint Committee on Cancer (AJCC) 7th edition)
3. Completion of an adjuvant oxaliplatin-based regimen, with no evident disease recurrence
4. Entry in the trial within 3 weeks of an adjuvant oxaliplatin-based regimen
5. Eastern Cooperative Oncology Group (ECOG) status of 0–2
6. Aged 20–80 years
7. Written informed consent provided to participate in the trial

### Exclusion criteria

Patients fulfilling any of the following criteria will be excluded from the trial:

- Previous or current systemic malignancy except curatively treated nonmelanoma skin cancer or in situ cervical carcinoma, unless there exists a DFS of at least 5 years
- Inadequate hematopoietic function defined as follows:
  1. Hemoglobin < 9 g/dL
  2. Absolute neutrophil count (ANC) < 1500/mm^3^
  3. Platelet count < 100,000/mm^3^
- Inadequate organ functions defined as follows:
  1. Total bilirubin >2 times the upper limit of normal (ULN)
  2. Hepatic transaminases (alanine aminotransferase (ALT) and aspartate aminotransferase (AST)) >2.5 × ULN
  3. Creatinine >1.5 × ULN
- Other severe medical conditions that are contraindicated to tegafur-uracil or render patients at a high risk of treatment complications based on investigator discretion
- Presence of other severe concomitant illness
- Participation in another clinical trial with any investigational product (IP) within 30 days before study entry
- Pregnant or lactating women or women of childbearing potential

### Trial enrollment method (Fig. 1,  Fig. 2)

**Fig. 1:**
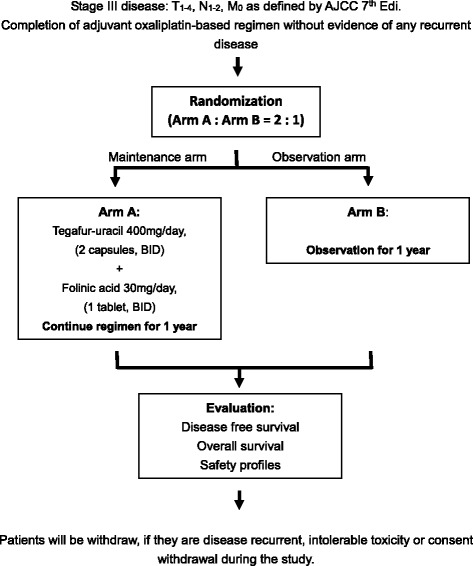
Trial enrollment. Stage III disease: T_1–4_, N_1–2_, and M_0_, as defined by the AJCC 7th edition. Completion of an adjuvant oxaliplatin-based regimen with no evident disease recurrence. The patients were withdrawn in cases of disease recurrence, intolerable toxicity, or consent withdrawal during the study

**Fig. 2:**
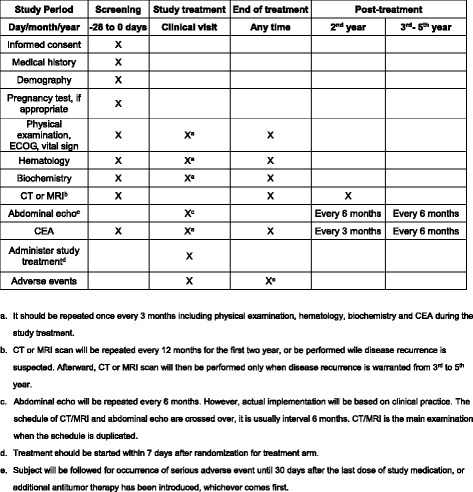
Flowsheet for pre/post-treatment investigations

Patient eligibility criteria will be established before treatment enrollment.

Following the completion of an adjuvant oxaliplatin-based regimen within 3 weeks with no evident disease recurrence, all eligible patients will be randomly assigned to either arm A (maintenance therapy) or arm B (observation arm) in a 2:1 ratio (364 and 182 patients in the tegafur-uracil and observation groups, respectively). Treatment in arm A will be started within 7 days of the randomization.

If the patients report disease recurrence, intolerable toxicity, or consent withdrawal or are removed according to the investigator’s discretion during the study period, they will be withdrawn from the study. If a patient wishes to discontinue the study, the corresponding data will not be reused, and the patient will not be allowed to re-enter the study.

## Study plan

### Study design

This is an open-label, randomized, comparative, double-arm, multicenter, phase III study to assess DFS, OS, and safety profiles with tegafur-uracil following an adjuvant oxaliplatin-based regimen as maintenance therapy for 1 year in patients with stage III colon cancer after radical resection in Taiwan (see Additional file 1 for the SPIRIT 2013 checklist).

### Patient number

Eligible patients will be randomized in two arms in a 2:1 ratio to reach an approximate total of 546 patients.

### Study schedule

Study date: the time of obtaining an approval letter from both regulatory authority and Institutional Review Board (IRB).

- Expected recruitment rate: 24 patients/month
- Recruitment date: at least 2 years
- Study duration: at least 5 years

### Visit schedule

The schedule of assessments (see ‘Assessment schedule’ below for details) indicates the number and timing of the planned visits, which must be followed as accurately as possible. Moreover, the visit schedule must be accurately followed.

### Treatment duration

Treatment will be administered for up to a maximum of 1 year, and in case of disease progression, intolerable toxicity, or consent withdrawal during the study, the patients will be withdrawn.

## Trial treatment

### Data on investigational product (Table 1)

**Table 1:** Data on the investigational product

Name of active ingredients	Tegafur	Uracil
Description	Tegafur occurs as a white crystalline powder. It is soluble in methanol, sparingly in water and ethanol, and slightly in ether. It is soluble in dilute sodium hydroxide.	Uracil occurs as a white crystal or crystalline powder, free of odor or taste. It is slightly soluble in water; slightly soluble in methanol, ethanol and acetone; and insoluble in ethyl acetate and chloroform.
Structure		
Chemical name	1-(2-tetrahydrofuryl)-5-fluorouracid 5-fluoro-1-(2-tetrahydrofuryl)-2,4 (1H,3H)-pyrimidinedione (IUPAC)	2,4(1H,3H)-pyrimidinedione
Molecular formula	C_8_H_9_FN_2_O_3_	C_4_H_4_N_2_O_2_
Molecular weight	200.17	112.09
Melting point	166 °C–171 °C	Approximately 335 °C
Contents	Each capsule contains 100 mg tegafur and 224 mg uracil.	
Storage condition	Below 30 °C	

#### Preparation and administration of the investigational product

Tegafur-uracil capsules (200 mg) will be administered twice daily (400 mg daily) at regular intervals. They will be administered whole and either 1 h before or after food consumption in the morning and evening.

### Labeling and supply

Tegafur-uracil is commercially available and supplied by the investigator’s pharmacy. The batch numbers of the used drugs will be diligently obtained and recorded.

### Storage

The drugs will be stored at below 30 °C. Procedures for the appropriate handling and disposal of anticancer medications will be followed.

### Dosage and treatment regimens

Eligible patients will receive orally administered tegafur-uracil (400 mg daily; 100 mg/capsule, two capsules each time, twice daily) and folinic acid (30 mg daily; 15 mg/tablet, one tablet each time, twice daily) for 1 year.

### Dose modification

- The dose after the first treatment will be modified on the basis of the highest toxicity degree graded according to National Cancer Institute Common Terminology Criteria for Adverse Events (NCI-CTCAE) v4.03
- For toxicities that are considered by the investigator to be unlikely to become severe or life-threatening and that do not result in a delay or interruption of therapy (e.g., alopecia and altered taste), treatment will be continued at the same dosing without reduction or interruption. No dose reductions or interruptions are required for anemia because it can be satisfactorily controlled by transfusions
- For the patients experiencing treatment interruption because of AEs for more than 2 weeks, treatment discontinuation will be considered (Table 2)

**Table 2:** Dose modification

Toxicity (grade)	Dose modification
ANC <1000/mm^3^	Discontinue the treatment until ANC ≥1500/mm^3^; resume the treatment with the same dose
Platelets <50,000/mm^3^	Discontinue the treatment until platelets ≥100,000/mm^3^; resume the treatment with the same dose
Other toxicities	No dose modification or interruption required
Grades 1 and 2	Discontinue the treatment until returns to baseline; resume the treatment to 200 mg daily
Grades 3 and 4
- *ANC* absolute neutrophil count
  - Discontinuation of study participation

If the patients experience any of the following consequence during the study, they will be withdrawn from the study treatment:

- Disease recurrence
- Intolerable toxicity
- Consent withdrawal
- Investigator discretion

All information regarding the withdrawal will be recorded in the Case Report Form (CRF)

### Prestudy, concomitant, and post-study treatments

1. Concomitant treatment
  The use of all concomitant medications and supportive therapies at baseline will be recorded throughout the study.
2. Prohibited treatment
  Treatment for cancer
  Except for the assigned study treatment, no other treatments known to exert an anticancer effect can be used during the trial (e.g., other chemotherapies, hormonal therapy, immunotherapy, antibody therapy, radiotherapy, thermotherapy, surgery, and IPs)
  Flucytosine
  Radiotherapy for supportive care
  Other IPs
3. Treatment with precaution
  Coumarin anticoagulants
  The patients treated with coumadin anticoagulants, such as warfarin, and concomitantly with tegafur-uracil will be regularly monitored for alterations in prothrombin time or the international normalized ratio
  Phenytoin
    The patients taking phenytoin concomitantly with tegafur-uracil will be regularly monitored for an increased phenytoin plasma concentration

## Study measurement and endpoints

### Screening and demographic measurements

The assessment schedule is illustrated in the ‘Assessment schedule’ section below. Each patient must sign and date an informed consent form before undergoing any study specific procedure, unless the procedure is considered the standard of care.

Screening procedures will be performed before the first treatment according to the assessment schedule within the required timeline.

1. Demographic and past medical history
  A complete medical history will be obtained at the screening visit. The medical history will include demographic information as well as any information pertinent to a CRC diagnosis
2. Physical examination, vital signs, and performance status
  Physical examination will be conducted, and vital signs and the performance status will be reported within 14 days before the first treatment and be repeated at every visit until study completion.
  The vital signs, including height, weight, blood pressure, and performance status, will be recorded according to the ECOG Scale
3. Hematological and biochemical tests
  Hematological and biochemical tests will be performed within 14 days before the first treatment. If any required hematological or biochemical values do not meet the eligibility criteria, the tests will be repeated before enrollment. If the tests performed within 7 days before the first treatment fulfill the eligibility criteria, tests are not required on day 1 of treatment.
  The hematological test comprises complete blood count (CBC) and differentiation count (D/C). CBC includes hemoglobin, hematocrit, erythrocytes, leukocytes, neutrophils, and platelets; The D/C will be calculated to obtain the ANC.
  The biochemical test measures albumin, alkaline phosphatase, ALT, AST, bilirubin (total and direct), blood urea nitrogen (BUN), and creatinine
4. Carcinoembryonic antigen
  Carcinoembryonic antigen (CEA) will be assessed every 3 months before the first treatment and will be repeated every 3 months in the first and second years and every 6 months thereafter
5. Computed tomography or magnetic resonance imaging
  Computed tomography (CT) or magnetic resonance imaging (MRI) will be annually performed in the first 2 years or if disease recurrence is suspected. The acceptable window for scheduling the imaging studies is ±1 week. Subsequently, CT or MRI will be performed only on suspected disease recurrence thereafter
6. Abdominal echo
  Abdominal echo will be performed every 6 months. However, its actual implementation will be based on clinical practice. The schedule of CT or MRI and abdominal echo are crossed over, and the typical interval is 6 months. CT or MRI is the main examination when the schedule is duplicated
7. Chest X-ray
  Chest X-ray will be annually performed
8. Adverse events
  AEs will be recorded from the first treatment to treatment completion

### Efficacy measurements and endpoints

In this study, we set 3-year DFS and 5-year OS as the primary endpoints and the differences the difference in survival distributions between two groups were analyzed using the log-rank test.

- Three-year disease-free survival (DFS)
  CEA and CT or MRI scan will be performed within 28 days before the treatment. The CEA level will be the efficacy measurement for endpoints and will be performed within every 3 months before the first treatment and repeated every 3 months in the first and second year and every 6 months thereafter. CT or MRI can be performed annually in the first 2 years or on suspected disease recurrence. The acceptable window for scheduling the imaging studies is ±1 week. Thereafter, CT or MRI will be performed only on suspected disease recurrence. DFS will be measured from start from the date of randomization to the date of disease recurrence
- Five-year overall survival (OS)
  OS will be measured from the start from the date of randomization to the date of death
- Safety profile
  Hematological and biochemical tests should be closely monitored during the treatment period because bone marrow depression, liver dysfunction, dehydration, anorexia, nausea, vomiting, and other AEs that have been reported in clinical treatment. The evaluation results would be included in the date or symptoms mentioned in the package insert

### Safety measurements and endpoints

- Hematology and biochemistry
  Hematological and biochemical assessments will be performed within 7 days before enrollment and at each clinical visit, as reported in Table 3. The clinically relevant findings will be recorded and graded according to NCI-CTCAE v4.03

**Table 3:** Flowchart for pretreatment and post-treatment investigations

Study period	Screening	Study treatment	Treatment completion	Post treatment	
Day/month/year	−28 to 0 days	Clinical visit	Any time	2nd year	3rd–5th years
Informed consent	X				
Medical history	X				
Demography	X				
Pregnancy test, if appropriate	X				
Physical examination, ECOG, vital signs	X	X^a^	X		
Hematology	X	X^a^	X		
Biochemistry	X	X^a^	X		
CT or MRI^b^	X		X	X	
Abdominal echo^c^		X^c^		Every 6 months	Every 6 months
Carcinoembryonic antigen (CEA)	X	X^a^	X	Every 3 months	Every 6 months
Study treatment^d^		X			
Adverse events		X	X^e^		

^a^It will be repeated once every 3 months, including the assessment of physical status, Eastern Cooperative Oncology Group (ECOG) status, vital signs, hematological and biochemical data, and CEA, during the study treatment

^b^Computed tomography (CT) or magnetic resonance imaging (MRI) will be repeated every 12 months for the first 2 years or on suspecting disease recurrence. Thereafter, CT or MRI will be performed only on suspected disease recurrence

^c^Abdominal echo will be repeated every 6 months. However, actual implementation will be based on clinical practice. The schedule of CT or MRI and abdominal echo are crossed over; the examinations are typically performed at an interval of 6 months. CT or MRI is the main examination used when the schedule is duplicated

^d^Treatment in arm A will be started within 7 days after the randomization

^e^The patients will be followed for the occurrence of serious adverse events (SAEs) until 30 days after the last dose of study medication or introduction of an additional antitumor therapy, whichever occurs first
- Other toxicities
  Any toxicities other than those observed in hematological and biochemical assessments will be recorded and graded according to NCI-CTCAE v4.03

## Assessment schedule (Table 3)

### Data management and statistical methods

#### Data management

CRFs will be provided for recording the data. Data will be recorded directly and legibly onto the CRFs by using a black-ink pen. If any data are unavailable, reason for omissions will be mentioned on the CRFs. Corrections will be made legibly and initiated and dated by an approved personnel; the reasons for marked changes will be provided. Correction fluid or covering labels will not be used.

#### Statistical methods and sample size determination

This is an open-label, randomized, comparative, double-arm, multicenter, phase III study of tegafur-uracil following an adjuvant oxaliplatin-based regimen in patients with stage III colon cancer after radical resection.

1. Statistical evaluation
2. Statistical analysis methods
  A comprehensive statistical analysis plan will be prepared before database lock. The patients will be categorized into the Full Analysis Set (FAS), per protocol (PP), and safety populations according to the following definitions:
  Full Analysis Set (FAS):
    The FAS is defined as containing all registered and randomized patients who received at least one dose of the study treatment without major protocol violations, such as noncompliance with the eligibility criteria, or other major violations (defined before database lock) during the study
  Intent-to-treat (ITT):
    The ITT population is the subset of the FAS population, and as all randomized patients irrespective of treatment received
  Per protocol (PP):
    The PP population is the subset of the ITT population and subjects who complete 12 months of tegafur-uracil treatment/observation with at least one biomarker assessment post treatment. The subject with confirmed early disease recurrence/relapse would be included in the PP population. The patients with confirmed early disease recurrence and relapse will be included in the PP population.
    All the efficacy endpoints will be analyzed in the FAS and PP populations. The main assessment will be performed on the basis of the PP population.
    The safety population is defined as all patients who are exposed to at least one dose of the study treatment and who are available for obtaining follow-up safety information
3. Statistical analyses
  Demographic and baseline measures
    All demographic and baseline data will be summarized and listed in detail. For continuous variables, the descriptive statistics, including the patient number, mean, median, standard deviation, and range (minimum-maximum) will be presented; frequency tables will be displayed for categorical data. If demographic and baseline measures are significant, their inclusion in the analysis model will be considered
  Three-year disease-free survival (DFS)
    Three-year DFS will be defined as the percentage of patients alive without disease recurrence at 3 years measured from the randomization date. The 3-year DFS will be evaluated using the Kaplan-Meier method, and the analysis will be presented in descriptive statistics, presented by a point estimate and 95% confidence interval. The results will be summarized in the form of the sample size, mean, standard error, median, percentiles, and range by descriptive statistical methods
  Five-year overall survival (OS)
    Five-year OS will be defined as the percentage of patients alive at 5 years measured from the randomization date. The 5-year OS will be evaluated using the Kaplan-Meier method, and the analysis will be presented in descriptive statistics, presented by a point estimate and 95% confidence interval. The results will be summarized in the form of the sample size, mean, standard error, median, percentiles, and range by using descriptive statistical methods
  Safety profile
    AEs will be summarized using NCI-CTCAE v4.03 and the preferred term. The incidence and percentage of patients with at least one occurrence of a preferred term will be included according to the most severe NCI-CTCAE v4.03 grade. Laboratorial toxicity will be presented according to the highest NCI-CTCAE v4.03 grade by cycle

### Adverse events

Timely, accurate, and complete reporting and analysis of safety information from clinical studies are crucial for the treatment of patients and for investigators and are mandated by regulatory agencies worldwide.

### Definitions

1. Adverse events
  An AE is defined as any untoward medical occurrence in a clinical study patient administered a pharmaceutical product, and it does not necessarily have a causal relationship with the treatment. Therefore, an AE can be any unfavorable and unintended sign (including an abnormal laboratory finding), symptom or disease temporally associated with the use of an IP, whether related to the product or not.
  Definition per International Conference on Harmonization (ICH)
  An AE is any occurrence that is new in onset or aggravated in severity or frequency from the baseline condition or abnormal results of diagnostic procedures, including laboratory test abnormalities
2. Serious adverse events:
  A serious adverse event (SAE), as defined by ICH, is any untoward medical occurrence that at any dose meets any of the following conditions:
  Results in death
  Is life-threatening (note: the term life-threatening in the definition of severe refers to an event, in which the patients are at a risk of death at the time of the event; it does not refer to an event, which hypothetically might have caused death if it were more severe)
  Requires inpatient hospitalization or prolongation of existing hospitalization
  Results in persistent or marked disability or incapacity
  Is a congenital anomaly or birth defect
  Requires medical intervention to prevent permanent impairment or damage
  Medical and scientific judgment will be exercised in deciding whether expedited reporting is appropriate in other situations, such as crucial medical events that may not be immediately life-threatening or result in death or hospitalization but may jeopardize patient health or require intervention to prevent one of the other outcomes listed in the aforementioned definition. These events will also typically be considered severe. Examples of such events are intensive treatment in an emergency room or at home for allergic bronchospasm, blood dyscrasias or convulsions that do not result in hospitalization, or development of drug dependency or abuse
3. Unlisted or unexpected adverse events
  The characteristic or severity of an unlisted or unexpected AE is not consistent with the applicable product information. For an IP, the expectedness of an AE will be determined by whether or not it is listed in the investigator’s brochure
4. Reversibility of adverse events
  AE disappearance is defined as an absence of AEs or recovery of AE severity to baseline values. AE alleviation is defined as the recovery of severity to grade 1 and is applicable for grade 2 or higher AEs only

### Reporting

1. Adverse event reporting
  All AEs will be reported from the time that a signed and dated Informed Consent Form is obtained until the completion of the last study-related procedure.
  All AEs, regardless of their seriousness, severity, or presumed relationship to the study therapy, will be recorded using medical terminology in the source document and CRF. Whenever possible, diagnoses will be performed if signs and symptoms are caused by a common etiology. Investigators will record in the CRF their opinions concerning the relationship of the AE to the study therapy. All measures required for AE management will be recorded in the source document
2. Severity and causality
  AE severity and relationship to the study medication will be evaluated and recorded in the CRF
  Severity categories (Table 4)

**Table 4:** Severity categories of adverse events (AEs)

Severity	Description
Mild	NCI-CTCAE v4.03 grade 1
	The AE does not limit daily activities; the patients may experience slight discomfort
Moderate	NCI-CTCAE v4.03 grade 2
	The AE results in some limitation of daily activities; the patients may experience considerable discomfort
Severe	NCI-CTCAE v4.03 grade 3
	The AE results in an inability to perform daily activities; the patients may experience intolerable discomfort
Life-threatening/disabling	NCI-CTCAE v4.03 grade 4
	The AE results in a disabling situation
	The AE results in a life-threatening situation
Death	NCI-CTCAE v4.03 grade 5

*The term life-threatening in the definition of severe refers to an event, in which the patients are at a risk of death at the time of the event; it does not refer to an event which, hypothetically, might have caused death if it were more severe (ICH-E2A)
  Causality to study medication The causal relationship with the study medication will be judged considering a patient’s systemic conditions, complications, concomitant medications, combination therapies, and temporal relationship. A reasonable possibility of the relationship between an AE and the study medication will be categorized into two categories: yes (presence of a reasonable causal relationship) or no (absence of a reasonable causal relationship; refer to the Council for International Organizations of Medical Sciences for definition of causality VI. If the investigator judges the relationship to be a “yes,” the event will be considered an adverse drug reaction (Table 5).

**Table 5:** Adverse drug reaction (ADR)

Unrelated	An adverse event (AE) that is not related to drug use
Unlikely	An AE for which an alternative explanation is more likely (e.g., concomitant drugs and diseases), or the relationship in time suggests that a causal relationship is unlikely
Possible	An AE that might be caused by drug use. An alternative explanation (e.g., concomitant drugs and diseases) is inconclusive. The relationship in time is reasonable; therefore, the causal relationship cannot be excluded
Probable or likely	An AE that might be due to drug use. The relationship in time is suggestive (e.g., confirmed by a dechallenge). An alternative explanation is less likely (e.g., concomitant drugs or diseases)
Certain	An AE that is listed as a possible ADR and cannot be reasonably explained by an alternative explanation (e.g., concomitant drugs and diseases). The relationship in time is highly suggestive; it is confirmed by a dechallenge and rechallenge
3. Serious adverse event reporting
  All SAEs occurring during clinical studies will be reported to the IRBs by the investigational staff within the timeframe required by individual IRBs.
  The cause of death of a patient in a clinical study, whether or not the event is expected or associated with the IP, is considered an SAE. Any event requiring hospitalization (or prolongation of hospitalization and exceeds a 24-h emergency visit) that occurs during a patient’s participation in a clinical study will be reported as an SAE, except hospitalizations for the following reasons:
  Social reasons in the absence of an AE
  Surgery or procedure planned before the study entry will be documented in the CRF Note: medical and scientific judgment will be exercised in deciding whether expedited reporting is appropriate in situations other than those listed above. For example, crucial medical events may not be immediately life-threatening or result in death or hospitalization but may jeopardize patient health or require intervention to prevent one of the aforementioned outcomes. Any AE is considered severe if it is associated with clinical signs or symptoms judged by the investigator to exert a marked clinical effect

### Follow-up

1. For safe follow-up, each patient will be followed for the occurrence of SAEs until 30 days after the last dose of the study medication or introduction of an additional antitumor therapy, whichever occurs first
2. All SAEs that have not been resolved by the end of the study or upon discontinuation of the patient’s participation in the study will be followed until any of the following occurs:
  The event is resolved
  The event is stabilized
  The event severity returns to baseline, if a baseline value is available
  The event can be attributed to agents other than the study medication or to factors unrelated to the study procedures
  When obtaining additional information is unlikely (the patients or health care practitioners refuse to provide additional information or the patients are lost to follow-up despite diligent follow-up efforts)

### Pregnancy report

Patient pregnancy will be reported by the investigational staff within 24 h of their knowledge. The study medication may affect pregnancies in partners of male patients included in the study; therefore, it will be reported by the investigational staff within 24 h of their knowledge. Any patient who becomes pregnant during the study will be promptly withdrawn from the study. Follow-up information regarding outcomes of the pregnancy and any postnatal sequelae in the infant will be reported.

### Good Clinical Practice

The trial will be conducted in accordance with the World Medical Association’s Declaration of Helsinki, as amended in 1996, and Good Clinical Practice (GCP) principles, as defined in the ICH of Technical Requirements for Registration of Pharmaceuticals for Human Use Harmonized Tripartite Guidelines for GCP.

### Institutional Review Board approval

The investigator will submit a copy of the protocol to the local IRB for consideration. The study will not start in each center until the IRB provides written approval of the protocol.

### Consent for publication

The investigator will be responsible for providing written (Patient Information Sheets) and verbal information to the patient and obtaining written informed consent before conducting study-specific procedures for the patients enrolled in the trial. Informed consent will be presented in language comprehensible to the patients or their legally authorized representatives. The patients or their representatives will be provided with adequate time to read the completed informed consent and their questions will be addressed. Informed consent will be obtained in a noncoercive manner, and the patients will be informed that participation is voluntary and will not affect the care that they may otherwise receive. After obtaining the consent, the patients or their representatives will be asked to sign and date the Consent Form, and the investigator or other authorized individuals obtaining the consent will also sign and date the form. A copy of the completed Consent Form will be provided to the patients. The investigator will confirm in writing that informed consent has been obtained and that a copy of the Consent Form is provided to the patients.

### Premature trial termination

The regulatory authority, IRB, and the investigator have the right to terminate the trial at any time for reasonable medical and administrative reasons. Reasons for termination will be appropriately documented.

## Discussion

CRC remains a leading form of cancer worldwide. In Taiwan, CRC is the most common cancer and the third leading cause of cancer-related deaths. The annual incidence of CRC in Taiwan has increased up to 43.5% in the past decade, with >15,000 new cases and >5000 deaths annually [1]. Randomized clinical trials have reported that the survival rate and quality of life in patients with stage III colon cancer who received chemotherapy were superior to those who were offered the most favorable supportive care alone. These observations support the use of different combination chemotherapy regimens. Moreover, novel therapeutic strategies are required to achieve more favorable clinical efficacy, with an acceptable toxicity profile.

Oxaliplatin, a platinum derivative, shows synergism; it is a third-generation diaminocyclohexane platinum compound that inhibits replication and transcription through the formation of DNA adducts [13]. To achieve more desirable efficacy and safety in stage III colon cancer, adjuvant chemotherapy with 5-FU has become the standard treatment. To provide the most favorable care for all patients with stage III colon cancer, aggressive treatment guidelines are crucial. Oxaliplatin, 5-FU, and LV combination chemotherapy is accepted and was reported as an effective first-line treatment regimen for advanced or metastatic gastric cancer or CRC [14, 15].

After radical surgery and adjuvant chemotherapy, more intensive treatment must be considered for stage III colon cancer to achieve more desirable disease control and improved OS. However, maintenance tegafur-uracil versus observation following an adjuvant oxaliplatin-based regimen in patients with stage III colon cancer after radical resection has not been highly altered in the last few decades. The efficacy and safety of chemotherapeutic agents are fundamental aspects of treating patients with stage III colon cancer. In this study, we set 3-year DFS and 5-year OS as the primary endpoints for evaluation to determine the effects of maintenance tegafur-uracil following an adjuvant oxaliplatin-based regimen in patients with stage III colon cancer.

In summary, adjuvant chemotherapy with oxaliplatin and tegafur-uracil is safe and has remarkable treatment efficacy, with the advantage of yielding a favorable response rate and a tolerable toxicity profile. We recognize that although observational studies provide some valuable information, they are not adequately capable of providing credible evidence. This observation, in addition to the limitations derived from the retrospective data collected from medical charts and lack of a control group, entails the urgent need to perform prospectively randomized studies to confirm the results.

Additional trials are required to confirm the benefits of tegafur-uracil administration following an adjuvant oxaliplatin-based regimen in patients with stage III colon cancer.

### Trial status

The trial started in July 2016 and is expected to enroll 546 patients in the study and control groups by the end of July 2018.

## Additional file

Additional file 1: Submission of a populated SPIRIT Checklist [16, 17]. This is an open-label, randomized, multicenter study of tegafur-uracil following adjuvant oxaliplatin-based regimen in patients with stage III colon cancer patients after radical resection. (PDF 53 kb)

## Abbreviations

5-FU: Fluorouracil
ADR: Adverse drug reaction
AE: Adverse event
AJCC: American Joint Committee on Cancer
ALT: Alanine aminotransferase
ANC: Absolute neutrophil count
AST: Aspartate aminotransferase
BUN: Blood urea nitrogen
CBC: Complete blood count
CEA: Carcinoembryonic antigen
CIOMS: Council for International Organizations of Medical Sciences
CRC: Colorectal cancer
CRF: Case Report Form
CT: Computed tomography
CTCAE: Common Terminology Criteria for Adverse Events
D/C: Differentiation count
DFS: Disease-free survival
DNA: Deoxyribonucleic acid
ECOG: Eastern Cooperative Oncology Group
FAS: Full Analysis Set
FdUMP: 5-Fluoro-2’-deoxyuridine monophosphate
FOLFOX: Folinic acid/fluorouracil/oxaliplatin
FUTP: 5-Fluorouridine triphosphate
GCP: Good Clinical Practice
ICH: International Conference on Harmonization
IP: Investigational product
IRB: Institutional Review Board
LV: Leucovorin
MOSAIC: The Multicenter International Study of oxaliplatin/5-fluorouracil/leucovorin in the adjuvant treatment of colon cancer
MRI: Magnetic resonance imaging
OS: Overall survival
PP: Per protocol
RNA: Ribonucleic acid
SAE: Serious adverse event
ULN: Upper limit of normal
UTP: Triphosphate
